# Missouri

**Published:** 1896-09

**Authors:** 


*Missouri.* St. Louis papers are decrying tuberculin as a diagnostic agent, because it did not prove a curative agent in human tuberculosis. Wake up, out there! Surely you do not want the East to think that the Western press has been sleeping the past five years and is ignorant of the advance of veterinary science in the direction of greater sanitary safeguards? Pull the scales off your eyes!

